# Thermal and impact histories of 25143 Itokawa recorded in Hayabusa particles

**DOI:** 10.1038/s41598-018-30192-4

**Published:** 2018-08-07

**Authors:** K. Terada, Y. Sano, N. Takahata, A. Ishida, A. Tsuchiyama, T. Nakamura, T. Noguchi, Y. Karouji, M. Uesugi, T. Yada, M. Nakabayashi, K. Fukuda, H. Nagahara

**Affiliations:** 1 Graduate School of Science, Osaka University, Toyonaka, 560-0043 Japan; 2 Atmosphere and Ocean Research Institute, The University of Tokyo, Chiba, 277-8564 Japan; 3 Graduate School of Science, Tohoku University, Sendai, 980-8578 Japan; 4 Graduate School of Science, Kyoto University, Kyoto, 606-8502 Japan; 5 Faculty of Arts and Science, Kyushu University, Fukuoka, 819-0395 Japan; 6 JAXA, Sagamihara, 252-5210 Japan; 7 JASRI/SPring-8, Hyogo, 679-5198 Japan; 8 Graduate School of Science, The University of Tokyo, Bunkyo-ku, 113-0033 Japan; 9 Department of Geoscience, University of Wisconsin-Madison, Wisconsin, 53706 USA

## Abstract

Understanding the origin and evolution of near-Earth asteroids (NEAs) is an issue of scientific interest and practical importance because NEAs are potentially hazardous to the Earth. However, when and how NEAs formed and their evolutionary history remain enigmas. Here, we report the U-Pb systematics of Itokawa particles for the first time. Ion microprobe analyses of seven phosphate grains from a single particle provide an isochron age of 4.64 ± 0.18 billion years (1σ). This ancient phosphate age is thought to represent the thermal metamorphism of Itokawa’s parent body, which is identical to that of typical LL chondrites. In addition, the incorporation of other particles suggests that a significant shock event might have occurred 1.51 ± 0.85 billion years ago (1σ), which is significantly different from the shock ages of 4.2 billion years of the majority of shocked LL chondrites and similar to that of the Chelyabinsk meteorite. Combining these data with recent Ar-Ar studies on particles from a different landing site, we conclude that a globally intense impact, possibly a catastrophic event, occurred ca. 1.4 Ga ago. This conclusion enables us to establish constraints on the timescale of asteroid disruption frequency, the validity of the crater chronology and the mean lifetime of small NEAs.

## Introduction

A long-standing issue in planetary science is the connection between individual meteorites and their asteroidal parent bodies. Based on telescopic observation, one of the largest groups of asteroids (S-type) was found to have a mineralogy (low-calcium pyroxene and olivine) similar to that of the most common class of meteorites (80%, ordinary chondrites)^1^. Recent visible and infrared spectroscopic measurements suggest that most S-type NEAs, including Itokawa, exhibit possible links to LL ordinary chondrites^2–4^. However, until the Hayabusa sample return mission, there was no direct evidence of a connection between meteorites and asteroids except the observed fall of Almahata Sitta, which provided a link between an F-type asteroid (2008 TC3) and dark carbon-rich anomalous ureilites^5^.

The Hayabusa sample-return mission was designed to explore a small S-type NEA, 25143 Itokawa, and has provided many insights into the properties of tiny “rubble-pile” NEAs (references in^6^). The initial comprehensive studies on the mineralogy, petrology, geochemistry and morphology of the regolith particles recovered from the Itokawa surface revealed that the particles consist of the same minerals as those that comprise LL5-6 chondrites (references in^7^). Based on two-pyroxene and/or olivine-spinel geothermometries, Nakamura *et al*.^7^ showed that the highly equilibrated particles experienced a peak metamorphic temperature of ~800 °C and cooled slowly to 600 °C. Since temperatures of thermal metamorphism in asteroids increase with depth, the observed maximum temperature of 800 °C and the slow cooling rate require a diameter of the original asteroid larger than 20 km, possibly with an onion-shell structure, such as the H chondrite parent body^8^. After this intense thermal metamorphism and the subsequent cooling, it is considered that the Itokawa parent body was catastrophically disaggregated by one or multiple impacts into numerous small pieces, some of which re-accreted into the present greatly diminished, rubble-pile asteroid^6,7^.

## Results

To better understand the thermal and impact histories of Itokawa, we investigated the U-Pb systematics of phosphate minerals in Itokawa regolith particles using an ion microprobe. Generally, phosphates are the primary carriers of U in ordinary chondrites and resistant to secondary thermal events due to their relatively high closure temperatures of 500–600 °C for Pb. One of the great advantages of U-Pb systematics is that there are two U decay series (^238^U-^206^Pb and ^235^U-^207^Pb), which potentially provides chronological information of not only a crystallization age but also an alteration age^9,10^. In this study, we adopt an advanced total Pb/U isochron method in the ^238^U/^206^Pb - ^207^Pb/^206^Pb - ^204^Pb/^206^Pb 3-D space for an improved age calibration from an obtained U-Pb data set. Cogenetic samples with an undistributed U-Pb system that share the same common-Pb isotopic composition must define a LINE in the ^238^U/^206^Pb - ^207^Pb/^206^Pb - ^204^Pb/^206^Pb space, whose intersection with the U-Pb concordia curve on the ^238^U/^206^Pb - ^207^Pb/^206^Pb plane provides a formation age. In contrast, for the discordia case, the plotted data in 3-D space are expressed by PLANAR regression. In this case, the upper and lower intercepts with the U-Pb concordia curve correspond to a formation age and an alteration age, respectively. We have demonstrated the robustness of our U-Pb *in situ* dating technique for various meteorites (references in^10^).

**Figure 1:**
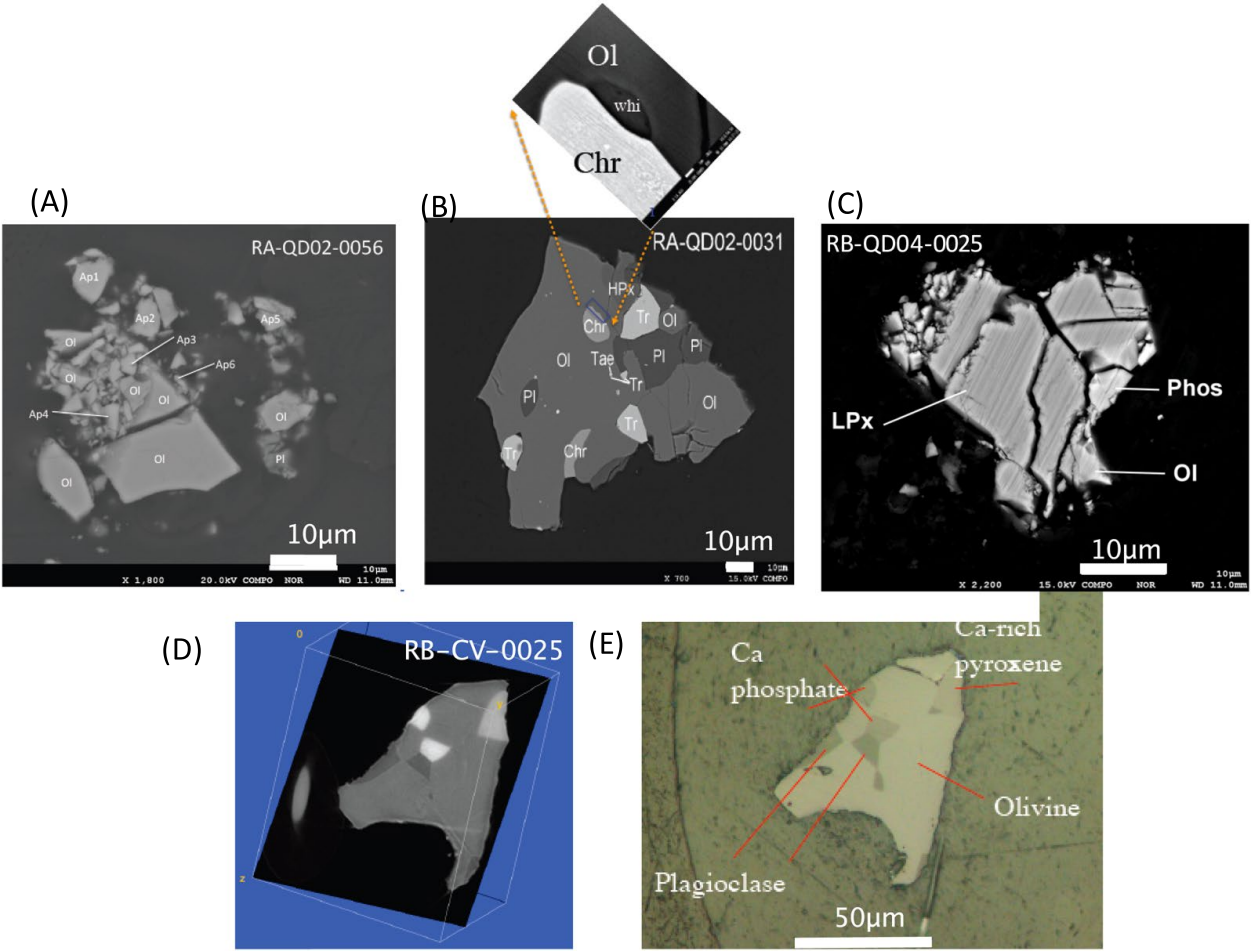
Back-scattered electron images of the Itokawa particles. (**A**–**C**) Show back-scattered images of polished sections of RA-QD02-0056, RA-QD02-0031, RB-QD04-0025, respectively. (**D**) Shows the “simulated” slice image of RB-CV-0025 before polishing, based on X-ray microtomography. Here, the angle and depth are selected where the cross sections inside two phosphates become the largest. (**E**) Shows the “actual” microscope image after careful manual polishing. Most phosphate grains are on the order of 2 µm × 4 µm to 4 µm × 5 µm in size.

Figure 1(A–C) show back-scattered images of allocated polished sections of RA-QD02-0056, RA-QD02-0031, RB-QD04-0025, respectively. For these grains, the phosphate locations are well confirmed based on SEM-EDS analysis. However, RB-CV-0025 was an entire particle (i.e., not polished) when the sample was allocated, although an analytical dual-energy X-ray microtomography^11^ suggests the occurrence of phosphates in the particle. Figure 1(D) shows the “simulated” slice image of the particle based on X-ray microtomography, where the cross sections inside two internal phosphates become as large as possible. Figure 1(E) shows the “actual” microscope image after careful manual polishing. RA-QD02-0031, RB-QD04-0025 and RB-CV-0025 exhibit a crystalline texture primarily consisting of olivine, plagioclase, chromite, pyroxene and troilite, with the accessary minerals taenite and whitlockite. In contrast, RA-QD02-0056 displays a brecciated texture and consists of olivine and plagioclase with several apatite grains. This particle is so fragile that there was no possibility of surviving the severe shock after compaction, which is well demonstrated by the fact that RA-QD02-0056 was split into small particles at the time of embedding in the epoxy resin. Most phosphate grains are from 2 µm × 4 µm to 5 µm × 7 µm in size. The chemical compositions of the phosphates are listed in Table 1. Based on the combination of two-pyroxene geothermometers, RA-QD02-0031 is an equilibrated particle that experienced a peak metamorphic temperature of 799 ± 16 °C. Although RA-QD02-0056 does not include two-pyroxenes, an Fa number for olivine of 29.6 ± 0.3 (n = 8) suggests that it is an equilibrium particle^7^. In previous studies, oxygen isotope measurements of olivine for both grains and the shock stage for RA-QD02-0031 have been investigated and exhibit similarity to less shocked LL chondrites^7,12^. Interestingly, it has been confirmed by TEM observation that RA-QD02-0056 consists of mineral grains whose surface has a thin layer (0–15 nm) induced by space weathering. Thus, the constituents of RA-QD02-0056 might have been individual particles before they formed a single grain possibly due to compaction.

**Table 1:** Chemical composition of typical phosphate grains.

Grain No.	RA-QD02-0056	RA-QD02-0056	RA-QD02-0056	RA-QD02-0031	RB-CV-0025	RB-CV-0025	RB-QD04-0025
Spot No.	1	2	3	1	1	2	1
SiO_2_	0.29	1.48	2.40	0.26	3.59	0.37	3.756
TiO_2_	0.06	n.d.	n.d.	n.d.	0.08	0.15	n.d.
Al_2_O_3_	n.d.	n.d.	n.d.	0.01	0.03	0.14	n.d.
FeO	0.10	0.26	1.12	1.18	n.d.	n.d.	2.592
MnO	n.d.	0.02	0.04	0.04	n.d.	n.d.	0.04
MgO	n.d.	0.47	2.13	3.56	7.58	4.02	7.79
CaO	55.60	53.12	50.67	42.31	40.53	45.36	40.871
Na_2_O	0.36	0.38	0.35	2.53	1.45	2.11	2.51
K_2_O	n.d.	0.03	0.02	0.07	n.d.	n.d.	0.049
Cr_2_O_3_	n.d.	n.d.	0.02	0.44	n.d.	n.d.	n.d.
NiO	0.02	n.d.	0.02	n.d.	n.d.	n.d.	n.d.
P_2_O_5_	42.80	40.11	38.67	42.69	37.32	42.32	39.589
SO_3_	0.07	0.11	0.04	0.06	0.01	0.01	n.d.
Cl	5.54	3.65	3.68	—	9.39	5.53	0.226
Total	103.59	99.45	98.66	93.15	99.98	100.01	97.382

**Figure 2:**
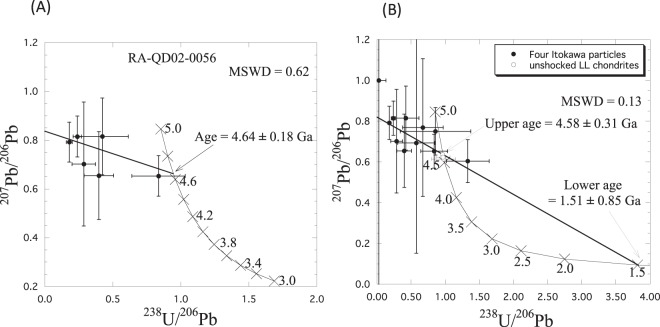
Result of *in situ* U-Pb dating of phosphates in Itokawa particles. The diagram projected onto the ^238^U/^206^Pb - ^207^Pb/^206^Pb plane of the total Pb/U isochron in three-dimensional ^238^U/^206^Pb - ^207^Pb/^206^Pb - ^204^Pb/^206^Pb space for a single particle (RA-QD02-0056) (**A**) and four particles (**B**), respectively. For comparison, the data for unshocked LL chondrites are shown in (**B**). The uncertainties of the plotted data and obtained ages are reported at the 1 sigma level. All data suggest that the crystallization ages of the Itokawa phosphates are approximately 4.6 Ga. U-Pb systematics of four Itokawa particles are well expressed by a planar regression in the 3-D space, providing an upper intersection age of 4.58 ± 0.31 Ga and a lower intersection age of 1.51 ± 0.85 Ga (1σ) with the concordia line on the ^238^U/^206^Pb - ^207^Pb/^206^Pb plane. These ages are thought to be a thermal metamorphism age and a shock age, respectively. The linear regression (**A**) and planar regression (**B**) were calibrated using Isoplot/Ex.

An ion microprobe study was performed on phosphates in four Itokawa particles with NanoSIMS at the University of Tokyo. The data are shown in Table 2. The large uncertainties of the individual data points are due to low counting rates. Specifically, data point #0031-01 (whitlockite) shows a large uncertainty because this grain is a particularly small whitlockite whose U concentration is extremely low (less than one-tenth that of apatite). The U-Pb systematics of a single particle (RA-QD02-0056) reveal it to be concordant, providing a total Pb/U isochron age of 4.64 ± 0.18 Ga (1σ) in the ^238^U/^206^Pb - ^207^Pb/^206^Pb - ^204^Pb/^206^Pb 3-D space (Fig. 2(A)). This outcome represents the first reported thermal metamorphism age of Itokawa particles. This age is identical to that of typical LL5 and LL6 chondrites (4.54–4.56 Ga)^13,14^. Although analytical uncertainty regarding the obtained U-Pb age is large, that ^26^Mg–excess was not observed in Itokawa particles^15^ suggests that thermal metamorphism continued for several million years (until ^26^Al decayed) after the CAI formation. The U-Pb systematics of RA-QD02-0031, which was not exposed to a severe shock (S2^12^ and/or up to S4^7^), are also consistent with those of RA-QD02-0056 within the analytical uncertainty. However, two other particles (RB−QD04-0025 and RB-CV-0025) exhibit discordant U-Pb systematics. Therefore, the combined U-Pb data of the four particles are NOT expressed by a linear regression in 3-D space but are well expressed by a planar regression (Y = −0.19X+ 0.82 + 0.37Z), providing an upper intersection age of 4.58 ± 0.31 Ga and a lower intersection age of 1.51± 0.85 Ga (1σ) (Fig. 2(B)). These outcomes are thought to be vestiges of a thermal metamorphism age and a shock age recorded in Itokawa particles, respectively. The younger age of 1.51 ± 0.85 Ga is consistent with Ar-Ar ages of (1.3–1.4) ± 0.3 Ga for three Itokawa particles^16^ but different from the Ar-Ar age of 2.3 ± 0.1 Ga for the single grain^17^. It should be noted that the U-Pb system is highly resistant to secondary events in contrast to the Ar-Ar system, which is sensitive to shock. Therefore, the observed U-Pb disturbance of Itokawa particles and the consistency of Ar-Ar ages of particles collected from a different landing site provide robust evidence that a cataclysmic/catastrophic impact event occurred on the Itokawa parent body 1.4 billion years ago (which is the weighted mean of the U-Pb discordant age and the Ar-Ar plateau age^16^), causing the disruption of the parent body of the Itokawa. Although the mineralogy and geochemistry of the Itokawa particles resemble those of LL chondrites^7^, this cataclysmic impact age differs from the Ar-Ar ages of most shocked LL chondrites, which exhibit a peak at approximately 4.2 Ga and no indication at approximately 1.3–1.4 Ga^18–20^. Considering the sensitivity of Ar-Ar ages to shock events, we conclude that Itokawa has experienced an evolutionary history different from that of most LL chondrites. However, certain LL chondrites, including the Chelyabinsk meteorite, exhibit evidence of a similar event near 1.5 Ga^21^. It is interesting to note that the Chelyabinsk meteorite is derived from the Flora and Baptistina asteroid families^22^ and that the estimated dispersion age of the Flora family (950 ± 200 Myr)^23^ is marginally consistent with our chronological records (1.51 ± 0.85 Ga for the U-Pb age and (1.3–1.4) ± 0.3 Ga for the Ar-Ar ages).

**Table 2:** Isotope ratios in phosphates for Itokawa particles and unshocked LL chondrites.

Grain No.	Spot No.	Mineral	U (p.p.m.)	^238^U/^206^Pb	^207^Pb/^206^Pb	^204^Pb/^206^Pb
RA-QD02-0056	0717_1	apatite	2.9	0.2405 ± 0.0310	0.8144 ± 0.0839	0.0270 ± 0.0079
RA-QD02-0056	0717_4	apatite	2.9	0.4241 ± 0.1913	0.8149 ± 0.1572	0.1251 ± 0.0487
RA-QD02-0056	0718_1	apatite	4.0	0.1809 ± 0.0216	0.7917 ± 0.0815	0.0298 ± 0.0073
RA-QD02-0056	0718_2	apatite	4.0	0.2878 ± 0.0859	0.7016 ± 0.2542	0.0302 ± 0.0340
RA-QD02-0056	0718_3	apatite	3.7	0.8375 ± 0.1959	0.6527 ± 0.0830	0.0007 ± 0.0022
RA-QD02-0056	0718_5	apatite	6.6	0.3992 ± 0.1080	0.6545 ± 0.1797	0.0128 ± 0.0179
RA-QD02-0031	0717_3	whitlockite	0.047	0.0266 ± 0.1047	1.0000 ± 1.0092	0.1133 ± 0.1931
RB-CV-0025	1218_1	whitlockite	0.201	0.6720 ± 0.2970	0.7690 ± 0.3380	0.0385 ± 0.0392
RB-CV-0025	1218_2	whitlockite	0.240	0.5760 ± 0.2710	0.6920 ± 0.5410	0.1540 ± 0.1170
RB-QD04-0025	0811_3	whitlockite	0.123	1.3301 ± 0.3132	0.6038 ± 0.1052	0.0755 ± 0.0391
RB-QD04-0025	0812_1	whitlockite	0.282	0.8570 ± 0.5160	0.7500 ± 0.1164	0.0357 ± 0.0263
from Göpel *et al*.^13^						
St. Severin (LL6)			0.44	0.96021 ± 0.02528	0.63590 ± 0.00041	0.00370 ± 0.00007
Guidder (LL5)			0.23	0.97578 ± 0.17499	0.61456 ± 0.00047	0.00069 ± 0.00009
Tuxtuac (LL5)			0.42	0.96693 ± 0.10284	0.62380 ± 0.00100	0.00195 ± 0.00014

## Discussion

Numerous chronological studies have been performed on S-type NEAs. Gladman *et al*.^24^ estimated the median lifetime of such NEAs to be ~10 Myr (up to 60 Myr). These NEAs terminate by striking a terrestrial planet (10–30% of NEAs), entering a Sun-grazing state (more than half) or escaping from the Solar System (approximately 15%). That NEAs exist in the vicinity of Earth suggests a continuous supply from the main belt to maintain the population of these objects with very short lifetimes compared to the age of the Solar System. Michel and Yoshikawa^25^ pointed out that most S-type NEAs, including Itokawa, likely originate in the inner main belt through the ν6 secular resonance. The dynamical time required to become an Earth-crosser through such a strong main belt resonance is estimated to be only a few million years^26^. In contrast, Bottke *et al*.^27^ reported asteroid disruption frequency in the main belt of once every billion years for objects >100 km and 15–30 times per billion years for objects 25–35 km in diameter. According to N-body calculations, the gravitational re-accumulation time after a collision is very short (typically several days)^28^. Using another approach, O’Brien *et al*.^29^ investigated the crater size distribution on the surface of several S-type asteroids observed at high spatial resolution by a spacecraft and reported 1 billion years for Gaspra (10–20 km size), 0.5–1 billion years for Ida (20–60 km size), and 1.2 million years for Eros (11–34 km). It should be noted that Gaspra and Ida lie in the main belt, but Eros is an NEA. Michel *et al*.^30^ recently suggested that the time required to accumulate Itokawa’s craters was at least 75 million years and maybe up to 1 billion years, depending on the applied scaling law. Tatsumi *et al*.^31^ also suggested a crater retention age of 3–33 Myr based on more realistic collision experiments^31^. Taking the probable size of the parent body of Itokawa (>20 km^7^) and/or LL chondrites (10–50 km^14^) into consideration, we can state that our radiometric impact age of 1.36 ± 0.24 Ga is approximately consistent with the timescale of asteroid collision frequency in the main belt and the crater chronology of S-type asteroids in the main belt and slightly longer than the crater age of Itokawa. This outcome may be the result of global resurfacing that resets the 1–10 m deep surface layer, which may have occurred in the main belt long after the possible catastrophic disruption of the rigid parent of Itokawa^31^. The short residence time of Itokawa particles (less than 8 Myrs), implied by estimation from cosmic-ray-produced ^22^Ne, also supports the recent resurfacing scenario (losing/cultivating its surface materials at a rate of approximately 10 m per 10^8^ years)^32^.

**Figure 3:**
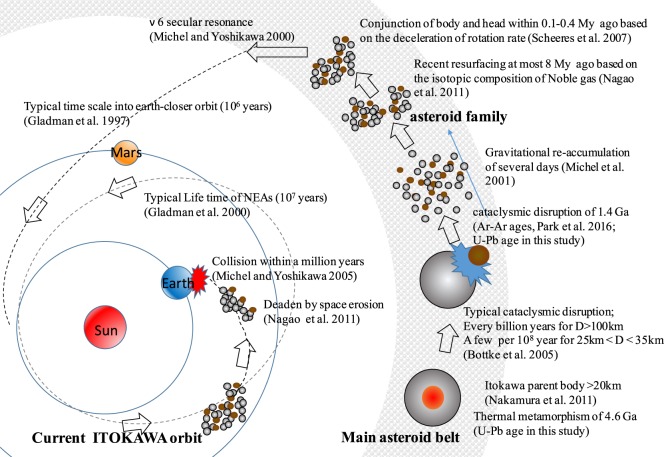
Overview of time evolution of the Itokawa asteroid. This illustration summarizes the various chronological data reported for the Itokawa asteroid, including evident radiometrical ages, the estimated timescale of N-body simulation and modelling based on the deceleration of the rotation rate (see text for details).

The observation of a deceleration in the rotation rate of Itokawa also constrains Itokawa’s evolution to within the last 0.1–0.4 million years during which period the conjunction of the two parts referred to as “head” and “body” by a low velocity impact occurred^33^. This conjunction must have occurred after the disruption event and the subsequent main re-accumulation and possibly while the Itokawa fragments still resided within the main belt. Combining our results with those of other theoretical studies, we propose that an intense impact event on Itokawa’s parent body (possibly catastrophic disruption) and reassembly occurred within the asteroid main belt and that its by-product, Itokawa, must have spent on the order of thousands of million years in the main belt. This period probably continued after the conjunction of the “head” and “body”, which belong to the “family”, within the last 0.1–0.4 million years. Then, Itokawa was injected into its current Earth-crossing orbit via the ν6 resonance. In addition, we predict that Itokawa will collide with the Earth within a million years^34^ and/or be destroyed by space erosion^32^. These chronological inferences for the Itokawa asteroid are summarized in Fig. 3.

Finally, we emphasize that our successful chronology results for Itokawa are based on a single brecciated particle, RA-QD02–0056, that includes several apatite grains. Such a brecciated grain is so fragile that it may not have been collected if the Hayabusa spacecraft sampling mechanism had operated as planned and its impactors struck the Itokawa surface. Fortunately, because the Hayabusa curation team found other particles that may include phosphorous minerals, further ion microprobe studies on the U-Pb systematics will place tighter constraints on local and global impact ages on Itokawa and clearly determine the time evolution of the typical small NEA Itokawa.

## Materials and Methods

Three polished sections of Itokawa particles (RA-QD02-0056, RA-QD02-0031, RB-QD04-0025) and one entire grain (RB-CV-0025) were provided by the Planetary Material Sample Curation Facility of JAXA (PMSCF⁄JAXA). For RB-CV-0025, analytical dual-energy X-ray microtomography^11^ suggested the occurrence of phosphates in the particle. Figure 1(D) shows the “simulated” slice image of the particle based on X-ray microtomography in which the cross sections inside two internal phosphates become as large as possible. Figure 1(E) shows the “actual” microscope image after careful manual polishing. After observation using a field emission scanning electron microscope at Kyoto University, the polished sections were gold-coated to prevent charging of the sample surface during NanoSIMS analyses. To further reduce the already very small ^x−1^PbH^+^ interference on the ^x^Pb^+^ peaks, the samples were baked and evacuated in the preload chamber overnight. In an important final step before the actual analysis, rastering of the primary ion beam over the sample surface for 5 minutes was performed to remove any remaining contaminants.

For *in situ* U–Pb dating, we used a NanoSIMS installed at the University of Tokyo, Japan. A 0.1 nA O^−^ primary beam with an acceleration voltage of 16 kV was focused to 1 µm and used to map an area of 2 µm × 2 µm to 5 µm × 5 µm by 32 pixels × 32 pixels depending on phosphate grain size. Then, positive secondary ions (^44^Ca^+^, ^204^Pb^+^, ^206^Pb^+^, ^207^Pb^+^, ^238^U^16^O^+^, ^238^U^16^O_2_^+^) were extracted and detected using the 5-Electron Multiplier (EM) system by 3-step magnetic-field switching as follows. In the first step, ^204^Pb^+^ ions were measured by a single collector. Then, ^44^Ca^+^, ^206^Pb^+^, ^238^U^16^O^+^, ^238^U^16^O_2_^+^ ions were simultaneously collected by four detectors. Finally ^207^Pb^+^ ions were measured by a single collector. The run tables of U-Pb dating in this study are listed in Table 3. One analysis session lasted approximately 180 minutes while switching the magnetic field (1 minute accumulation * 3 magnet steps * 60 cycles). The mass resolution was set at approximately 4200 at ^206^Pb (10% peak height), which is sufficient to separate Pb peaks from any interference peaks. A typical background is a few counts per 60 minutes per detector.

**Table 3:** Run table of U-Pb dating for phosphates.

Magnet field	EM1	EM2	EM3	EM4	EM5
B1			^204^Pb^+^		
B2	^44^Ca^+^		^206^Pb^+^	^238^U^16^O^+^	^238^U^16^O_2_^+^
B3			^207^Pb^+^		

Since these mapping areas are comparable and/or slightly larger than the actual grain size, we extracted the appropriate pixels from the mapping area, in which the ^44^Ca ion counts are larger than 90% of those of standard apatite, and accumulated secondary ion counts of these appropriate pixels. The mass resolution was set to approximately 4200 at ^206^Pb for the U–Pb analyses.

The abundance ratios of ^238^U to ^206^Pb for phosphates were calibrated from the observed ^238^UO^+^/^206^Pb^+^ ratios using an empirical quadratic relationship between the ^206^Pb^+^/^238^UO^+^ and ^238^UO_2_^+^/^238^UO^+^ ratios of standard apatite derived from an alkaline rock of the Prairie Lake circular complex in the Canadian Shield (1156 ± 45 Myr (2σ)^35^).
